# Effectiveness of the addition of Lidocaine to a hemostatic, bioresorbable putty in the treatment of iliac crest donor site pain

**DOI:** 10.1186/1471-2474-15-415

**Published:** 2014-12-08

**Authors:** Marc Andreas Müller, Arne Mehrkens, Roman Zürcher, Patrick Vavken, Victor Valderrabano

**Affiliations:** Orthopedic Department, University Hospital of Basel, University of Basel, Spitalstrasse 21, 4031 Basel, Switzerland; Department of Anesthesiology, University Hospital of Basel, Spitalstrasse 21, 4031 Basel, Switzerland; Department of Orthopedic Surgery, Children’s Hospital Boston, Harvard Medical School, 300 Longwood Avenue, Boston, MA 02215 USA; Center for Population and Development Studies, Harvard School of Public Health, 330 Brookline Avenue, Boston, MA 02215 USA

## Abstract

**Background:**

The harvest of iliac crest bone grafts (ICBG) is associated with relevant donor site pain, but may be lowered by the application of lidocaine loaded on biodegradable, hemostatic putty for sustained local analgesic release. The goal of this double-blind controlled trial was to assess the efficacy of adding lidocaine to a hemostatic putty (Orthostat ™) to treat donor site pain following harvest of ICBG in foot and ankle procedures.

**Methods:**

After ICBG harvest during a foot and ankle procedure, the resulting bone defect was either filled with Orthostat™ (n = 7) or with the same hemostatic putty loaded with lidocaine (Orthostat-L™, n = 7). During the first 72 postoperative hours, donor site and surgical site pain were managed by patient controlled morphine delivery and a peripheral nerve block. Donor site pain was periodically quantified on a Visual Analog (VAS) and a Wong Baker FACES scale. Pain scores were plotted over time to calculate the area under the curve (AUC) to quantify the overall pain experienced in specific time intervals.

**Results:**

Orthostat-L™ significantly reduced donor site pain over the first 12 hours postoperatively as evidenced by a significant decrease of the AUC in both VAS (p = 0.0366) and Wong Baker FACES pain score plots (p = 0.0024). Cumulated morphine uses were not significantly decreased with Orthostat-L™.

**Conclusion:**

The addition of lidocaine to a hemostatic putty offers a significant ICBG donor site pain reduction over the first 12 postoperative hours.

**Trial registration:**

ClinicalTrials.gov NCT01504035. Registered January 2nd 2012.

**Electronic supplementary material:**

The online version of this article (doi:10.1186/1471-2474-15-415) contains supplementary material, which is available to authorized users.

## Background

Cortical autologous iliac crest bone grafts (ICBG) are widely used in orthopedic surgical procedures[1], such as open wedge osteotomies[2–4], interposition arthrodeses[5, 6] and bone defects. Autologous bone grafts display excellent biological properties – osteoconductivity[7, 8], osteoinductivity[9], and potentially osteogenicity[10],- plus favorable biomechanical characteristics[11], and are considered the gold standard despite the abundance of allogenic and synthetic substitutes available[12]. However, these favorable characteristics have to be juxtaposed with the risk of associated donor site morbidity, which may be clinically relevant in approximately 5% of patients, with values up to 30% in some reports[13–18].

Patients undergoing ICBG harvest often experience significant post-surgical bleeding[14] and pain[17] at the harvest site and may rarely sustain more severe complications including fractures[16], arteriovenous fistula[14], nerve injuries[15] and abdominal wall hernias[19]. The administration of hemostatics in combination with local anesthetics has been proposed to manage pain and bleeding. However, local anesthetics may clear rapidly from the harvest site, potentially causing systemic toxicity and reducing duration and effectiveness of postoperative analgesia[20].

These considerations prompted the incorporation of lidocaine into a biodegradable hemostatic putty (Orthostat™ currently marketed as Hemasorb™) to locally provide anesthetic to the surgery site (Orthostat-L™). The Orthostat™ putty is composed of a polyvalent salt of a high molecular weight carboxylic acid, an alkyl benzopyranol ester and Vitamin E which enables a continuous release of lidocaine. Orthostat-L™ additionally comprises lidocaine at a concentration of 16% of its dry weight. In a previous in-vitro and animal study, lidocaine was released from the putty in a exponential fashion with a half-time of approximately 6–8 hours.[21].

With the addition of Lidocain to Orthostat, pain control may be provided by two mechanisms. The hemostatic component may decrease the formation of irritating subperiostal hematomas, while the continuous release of lidocaine may decrease pain perception at the periosteal level.

In animal studies, the application of Orthostat-L™ resulted in a dose dependent, sustained analgesia in rats over a duration of two to three days with no evidence of systemic toxicity[21–23]. However, to our knowledge no studies have been performed to confirm this in humans.

It was the primary objective of this study to compare the analgesic effectiveness of a lidocaine loaded hemostatic putty (Orthostat-L™) with a lidocaine deficient putty (Orthostat™), defined as visual analog (VAS) and Wong Baker FACES scale outcomes, in a random sample of patients undergoing ICBG harvest for foot and ankle procedures. We hypothesized that the addition of lidocaine to the putty would provide significantly better pain control in the early postoperative period. For the purpose of this study, we defined “early postoperative” as an observation period of 72 hours during which we searched for differences in reported pain.

As a second objective, we aimed to test if, after the administration of the lidocaine loaded hemostatic putty, peripheral blood licocaine levels would stay below the threshold for liver toxicity of 6 mg/l.

## Methods

### Study design

This study was designed as a prospective, comparative, active-controlled, double-blind trial of the analgesic effectiveness of Orthostat-L™ (experimental group) with Orthostat™ (active control) in patients undergoing iliac crest bone graft (ICBG) harvest. The main outcome measurement was pain measured on a VAS and Wong Baker scale. We aimed at being able to detect an at least 10% ± 5% difference between two independent groups in the cumulative VAS scores within the early postoperative time period with a power of no less than 90% and a two-tailed alpha of 5% . This is consistent with an effect size of 2, thus requiring a total sample size of 14, or 7 per group, to reach a power of 90%. Such a sample size would still allow detecting a sample size of 1.5 without less than at least 80% power.

### Study subjects

From May to July 2008, we enrolled 14 patients undergoing foot and ankle surgical procedures with the use of a structural iliac crest bone graft. Patients who were between 18 and 71 years old and capable of completing a patient administered analgesia (PCA) device and who had given their written informed consent were included in the study. Patients with previous ICBG harvest, serious medical conditions including liver and heart failure, bleeding diathesis, hypersensitivity to lidocaine or to components of the hemostatic putty or any mental (dementia/psychiatric disorder) impeding their cooperation in the postoperative evaluations were excluded. Upon inclusion, patients were randomly allocated to the Orthostat™ or Orthostat-L™ group. There were seven patients in each group. Patients as well as all medical staff were blinded to which product the patient received.

As a result of the random allocation, there were seven patients in each group. Table 1 lists the demographic characteristics of patients allocated to the Orthostat™ and Orthostat™-L group.

**Table 1:** **Patient demographics and intraoperative data (Data presented as mean ± SD)**

	Orthostat-L™ (n = 7)	Orthostat™ (n = 7)	P-value
Gender	4 Females, 3 Males	3 Females,4 Males	
Mean age (years)	42.6 ± 10.5	51.0 ± 16.9	0.248
Mean duration of Surgery (minutes)	169.4 ± 53.3	140.9 ± 38.5.	0.273
Graft size (cm^3^)	4.0 ± 2.9	3.4 ± 1.9	0.681
Putty amount (grams)	4.3 ± 1.2	4.3 ± 0.8	0.999
Mean time to hemostasis after putty administration (seconds)	24 ± 11	37 ± 37	0.392
Intraoperative blood loss at the ICBG harvest site (ml)	15 ± 8	15 ± 5	0.999

The study was conducted in accordance with the current version of the Declaration of Helsinki and under the laws and regulations enforced by the local ethics committees. This trial was registered in ClinicalTrials.gov (registry number: ClinicalTrials.gov NCT01504035, registered January 2nd 2012) and approved by the local ethical committee (EKBB registry number 14/08).

### Application of a continuous popliteal sciatic nerve block

Prior to induction of anesthesia, a board certified anaesthetist (RZ) administered a peripheral nerve block in the popliteal fossa proximal to the surgical site. Blocks were performed with 20–30 ml of mepivacaine 1.5% and bupivacaine 0.5%. After injection a 20G catheter was inserted for continuous postoperative sciatic nerve block with ropivacaine 0.2%, using an elastomer pump with a flow rate of 6-10 ml/h.

### Iliac crest bone graft (ICBG) harvest and application of Orthostat™/Orthostat-L™

Upon exposure and preparation of the primary surgical site at the foot and ankle, the same surgeon (VV) obtained an iliac crest bone graft in the following manner. A skin incision was made over the anterolateral aspect of the iliac crest. The aponeurosis of the external oblique was exposed and incised. The iliac crest was exposed subperiosteally, and a tricortical ICBG was obtained with an oscillating saw. The size of the graft was adapted to the structural defect at the foot and ankle to be bridged but was not larger than 3 cm × 1 cm × 2 cm (6 cm^3^). The graft dimensions were measured and the donor site was dried with sterile gauzes. Then either Orthostat™ or Orthostat-L™ was administered to the exposed bone surfaces under firm digital pressure until the level of material filling ICBG harvest site was flush with the bone surface. The amount of Orthostat™/Orthostat-L™ was determined by the number of 2 g portions applied to the harvest site and by weighing the leftover of the last portion used. The time from the end of putty application to complete hemostasis at the donor site was recorded and judged to be complete when there was no active bleeding noted by the surgeon. In addition the aspirated blood volume from the iliac crest was measured and recorded for blood loss. The wound was then closed in layers. No blood drain was inserted at the iliac crest harvest site.

### Outcome assessment - donor site pain

Patients were closely followed every four hours within the first 72 hours of Orthostat™/Orthostat-L™ administration by blinded assessors. The pain level at the pelvic harvest site was rated according to the VAS score and Wong-Baker FACES Pain Rating Scale. VAS scores were measured on a continuous scale ranging from 0–100 mm. The Wong Baker scale ranged from 0 to 5 points, with the verbal anchors “no pain” to “worst pain”. The VAS and Wong-Baker pain scores for each patient were plotted over time. The area under the curve (AUC) was calculated to represent the *cumulative* pain experienced within a specific time period. Since the surgical site pain from the lower extremity was addressed by a continuous sciatic nerve block, any pain experienced by the patient was related to iliac crest donor site pain. In case of any dysfunction of the sciatic block, the level of surgical site pain had to be recorded in the protocol.

Additional postoperative pain control was provided for all patients using patient controlled analgesia pumps (PCA) loaded with morphine. The cumulative dose of morphine used within the first 72 h after surgery was quantified.

### Outcome assessment - serum Lidocaine levels and adverse events

Serum lidocaine levels were measured after the administration of the peripheral nerve bloc (baseline) and at 2, 4, 8 12, 48, and 72 hours after the application of the putty using high performance liquid chromatography. Blood samples for serum lidocaine measurements were obtained from the basilic vein of the ipsi-contralateral arm and analysed immediately*.* The formation of a local hematoma at the iliac crest harvest site was assessed by ultrasound performed after 48–72 hours. After discharge from the hospital, patients were followed up at 14 and 30 days after surgery. During these follow-up visits, any adverse event such as prolonged pain, hematoma, herniation etc. at the pelvic harvest site was recorded.

### Statistical analysis

Two-tailed t-tests for independent samples were used to compare cumulative PCA, bleeding time, total blood loss, and graft size across treatment groups. Continuous variables including VAS pain scores at different time points as well as the area under the curve for VAS and Wong Baker scores were tested using repeated measure ANOVA. For the 16 hours’ time point, more than 20% of VAS and Wong Baker FACES measurements were missing and thus the time point had to be dropped. Discontinuous variables including Wong - Baker scores were analysed using Fisher’s exact test with Bonferroni adjustments for multiple measurements. Results are given as mean ± SD. An alpha of 5% was considered significant. All analyses were done using intercooled STATA 10 (Stata Corp LP, College Station, TX).

## Results

### Intra-operative data

As shown in Table 1, the duration of surgery, intraoperative blood loss and time to hemostasis after putty administration, as well as graft sizes and amount of putty used were not significantly different between Orthostat-L™ and Orthostat ™ (control) group.

### Analgesic effectiveness of Orthostat-L™ - donor site pain

**Figure 1:**
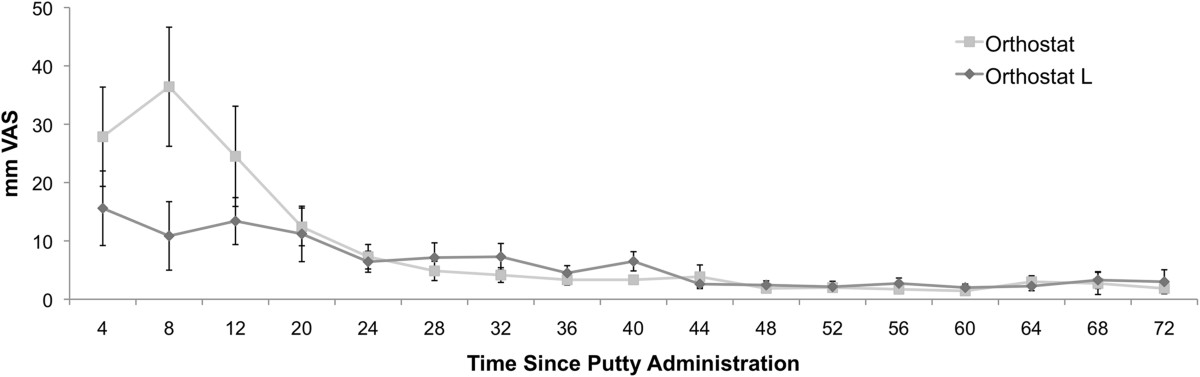
**Postoperative VAS pain scores recorded at periodical time intervals after putty administratioon.**

There were no cases of sciatic block dysfunction. All pain recorded was related to the iliac crest donor site. Subjects treated with Orthostat-L™ showed a significantly improved area under the curve (AUC) of the VAS score (AUC_vas_) as compared to Orthostat™ from 1 to 12 hours (mean AUC_vas_ 110 ± 115 vs. 291 ± 270; p = 0.036) (Figure 1). From 12 to 24 hours the control scores decreased towards baseline and differences between Orthostat-L™ and Orthostat™ were no longer statistically significant (AUC VAS 64 ± 62 vs 87 ± 79, p = 0.281). In the repeated measure ANOVA of all time points individually, there was no significant difference across groups VAS score (p = 0.3644) over the total follow-up.

**Figure 2:**
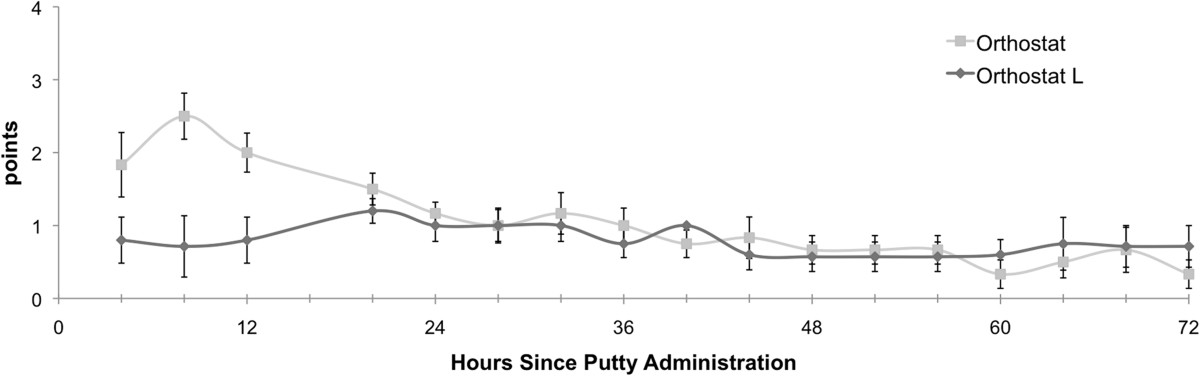
**Postoperative Wong-Baker FACES pain scores.**

For the Wong-Baker FACES Pain Rating Scale there was a significantly better outcome for Orthostat-L™ compared to Orthostat™ for the AUC for the first 12 hours (AUC _Wong-Baker_ 6 ± 6 vs. 15 ± 15, p = 0.0024 (Figure 2)) suggesting a beneficial early effect. Between 12 and 24 hours postoperatively as well as beyond 24 hours, as the control scores returned to baseline, the AUC obtained from Wong-Baker FACES Pain Rating Scale was not significantly different across groups (p = 0.999 and p = 0.1772, respectively), suggesting that the early effect equilibrates by the third postoperative day. There was no significant difference across groups regarding Wong-Baker FACES scores (p = 0.3544) at individual time points. There was no significant difference in cumulative PCA delivered morphine dose at 12 h (p = 0.267), 24 h (p = 0.717), or 72 hours postoperatively (p = 0.109) between the two groups.

### Safety of Orthostat-L™ - Lidocaine levels and adverse effects

**Figure 3:**
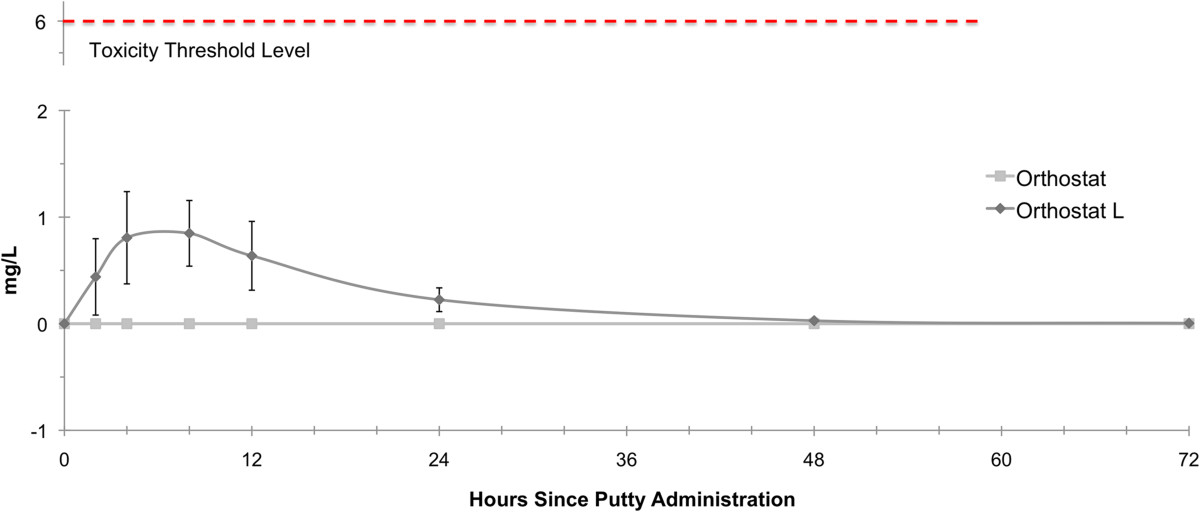
**Postoperative Serum lidocaine levels.**

In the Orthostat-L™ group, the serum levels of lidocaine peaked at 8 hours remained elevated until 40–48 hours. At all time points, lidocaine levels stayed well below toxic levels of 6 mg/l. The mean lidocaine levels in the peripheral blood was significantly higher (p = 0.022) for the Orthostat-L™ group than the control group (Figure 3).

There were no serious adverse effects experienced by subjects in the study. There was one case of seroma at the graft site in the intervention group that became evident prior to discharge on the fifth day after surgery. It required manual expression of the serous fluid to resolve. There were no complications in the control group (p = 0.2994).

## Discussion

Iliac crest bone graft harvesting is associated with a donor site morbidity of up to 30%[13, 14, 16]. Fortunately, major complications occur only rarely. However, patients frequently experience significant pain at the harvest site after ICBG harvest[17]. Standard pain management includes use of opioid analgesics, which may cause respiratory depression[24], nausea, vomiting or decreased gastrointestinal motility[25], and thus may temporarily worsen the patient’s condition and result in unfavorable outcomes and extended hospital stays with increased costs. Hence, reduction of postoperative pain at the harvest site will reduce or eliminate the comorbidities associated with use of systemically applied analgesics such as opioids. In this study we evaluated the effectiveness of a lidocaine loaded hemostatic putty (Orthostat-L™) as compared to a lidocaine deficient putty to lower iliac crest donor site pain.

Our study has potential limitations. Our sample was small, aiming for a between group difference of 10% ± 5%, and a larger study would be capable of detecting a smaller difference than that with statistical significance. However, the statistical significance of such a smaller difference must be put into relation with its clinical meaning, which might be questionable. Also, experiencing postoperative pain and response to analgesic medication has considerable inter-individual variability. Another potential shortcoming of a small sample size is a potentially higher risk of bias due to differential distribution. However, the enrolled patients were not significantly different in demographics and duration of procedure undergone, and systemic disease and lidocaine-related issues were exclusion criteria in our study. Hence it is not likely that our data are affected by substantial bias.

In our study, Orthostat-L™ significantly reduced cumulative VAS and Wong Baker FACES pain scores at the harvest site within the first 12 postoperative hours. Thereafter, pain scores in the control group began to return to baseline values. Nonetheless, pain scores in the Orthostat-L™-group continued to be lower compared to the Orthostat™-group, however, without statistical significance. The limited duration of significant pain relief provided by Orthostat-L™ may be explained by the fact that the control subjects returned to low pain levels just after 12 hours and by 20 hours after putty administration. Thus, there was no relevant pain for Orthostat-L to mitigate after this time period. In addition, the effects of lidocaine could have been also been altered by a local inflammatory response due to the surgical intervention and, additionally, to the putty. It has been postulated that inflammatory cell released peroxynitrite could decrease the effect of local anesthetic effects[26, 27]*.* Our data do not suggest that the duration of pain relief was restricted by limited lidocaine release from the putty. Lidocaine levels remained elevated in the Orthostat-L™ group even beyond 36 hours which was in fact in line with previous in vitro results[21]. Thus, Orthostat-L™ showed a much more favorable drug elution profile as compared to single administrations of local anesthetics. After single infiltration of the iliac crest with local anesthetics, plasma levels peak within 20–100 minutes and rapidly decrease thereafter[20].

Throughout the study, we could not detect a significant difference regarding cumulative PCA use between the two study groups. This result may be interpreted in two ways: First, our study was primarily powered to detect significant differences regarding cumulative VAS scores and *not* narcotic use. Second, Ip et al.[28] showed in a systematic review that there are determinants of postoperative pain medication use which are independent from VAS rated pain. These parameters include anxiety and behavioral abnormalities which were not assessed in our study. We can therefore not exclude any confounding by these psychological factors.

Our data do not suggest that the duration of pain relief was restricted by limited lidocaine release from the putty. Lidocaine levels remained elevated in the Orthostat-L™ group even beyond 36 hours which was in fact in line with previous in vitro results[21]. Thus, Orthostat-L™ showed a much more favorable drug elution profile as compared to single administrations of local anesthetics. After single infiltration of the iliac crest with local anesthetics, plasma levels peak within 20–100 minutes and rapidly decrease thereafter[20].

In terms of safety, our study was able to demonstrate that serum lidocaine levels remained well below the level of toxicity (6 mg/l). The maximum serum lidocaine level noted in our study was 2.1 mg/l and was registered after 4 hours. We noted the occurrence of one seroma in the Orthostat-L™ group. This complication may be rather interpreted as a minor donor site complication and not as an adverse event directly related to the local drug application. Such minor complications may occur with a frequency of 10-15%[14, 16].

Previous studies have already suggested the installation of local anesthetics to reduce donor site pain at the iliac crest[20, 29–35]. The proposed techniques vary from local infiltration at the time of surgery[20, 32, 35] to intermittent[30] or continuous[31, 33, 34] administration using indwelling catheters. Similar to our study, single or intermittent administration of local anesthetics at the harvest site significantly reduced donor site pain 12–24 hours postoperatively[20, 30, 35]. Serum concentrations also stayed below the toxic level[20]. Continuous administration could increase the duration of pain relief to up to 48 hours in one study[34] while another study could not demonstrate this effect[31]. Study results were also very conflicting regarding the effect on local anesthetics on the intake of narcotics in the postoperative period. Some studies showed that the application of local anesthetics at the iliac crest could significantly decrease the postoperative opioid demand[30, 34], while others could not reproduce these results[20, 31]. However, the aforementioned studies contained heterogeneous sample groups and showed variable confounders or biases. Most importantly, these studies did not eliminate the surgical site pain. Pain perception at one side increases anxiety and thus alters the pain perception across other body sites[36]. Accordingly, Morgan et al.[31] could demonstrate in their study a significant correlation between recipient and donor site pain. However, only few studies incorporated the level of pain at the surgical site in the analysis and interpretation of their data[20, 31, 34]. Our study could avoid these confounding effects since surgical site pain was continuously eliminated by the popliteal blocks. Thus, pain relieve at the donor site can more directly be attributed to the effectiveness of Orthostat-L™.

When comparing Orthostat-L™ with local anesthetics, it must be considered that that Orthostat-L™ is also a hemostatic agent. In contrast to local anesthetics, Orthostat-L™ may therefore additionally help to prevent irritating subperiosteal hematomas, which further contribute to donor site pain. Nevertheless, these speculations on the superior effectiveness of Orthostat-L™ to reduce donor site pain needs clarification in future randomized controlled trials.

## Conclusions

A bioresorbable hemostatic putty with lidocaine, as Orthostat-L™ has significant effectiveness in controlling pain experienced within 12 hours after iliac crest bone graft harvesting compared to a putty without lidocaine, as Orthostat™. We found no evidence for systemic toxicity or an increase in risk of adverse effect using a lidocaine based putty.

## Authors’ information

Marc Andreas Müller and Arne Mehrkens first authorship.

## Authors’ original submitted files for images

Below are the links to the authors’ original submitted files for images. Authors’ original file for figure 1 Authors’ original file for figure 2 Authors’ original file for figure 3
