# Supplementary figures and images for: Effectiveness of the addition of Lidocaine to a hemostatic, bioresorbable putty in the treatment of iliac crest donor site pain
Source: BMC Musculoskelet Disord. 2014 Dec 8;15:415. doi: 10.1186/1471-2474-15-415 (PMC4295296; doi:10.1186/1471-2474-15-415)

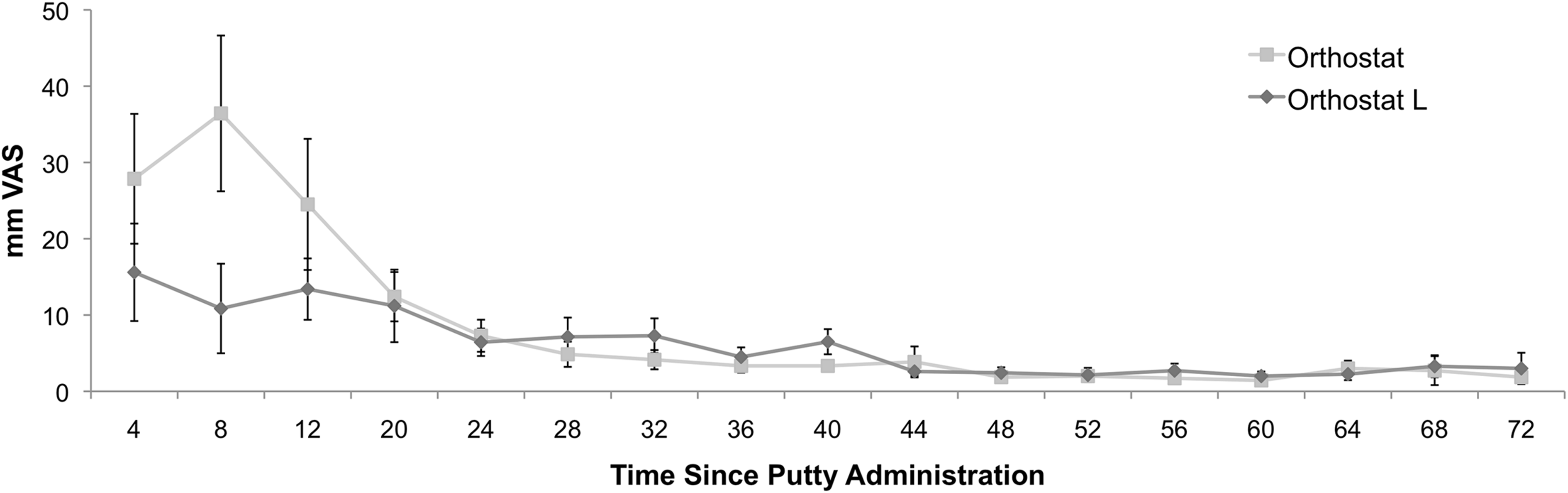

Supplement: Supplementary file 1 — Authors’ original file for figure 1 [file 12891_2013_2349_MOESM1_ESM.tiff]

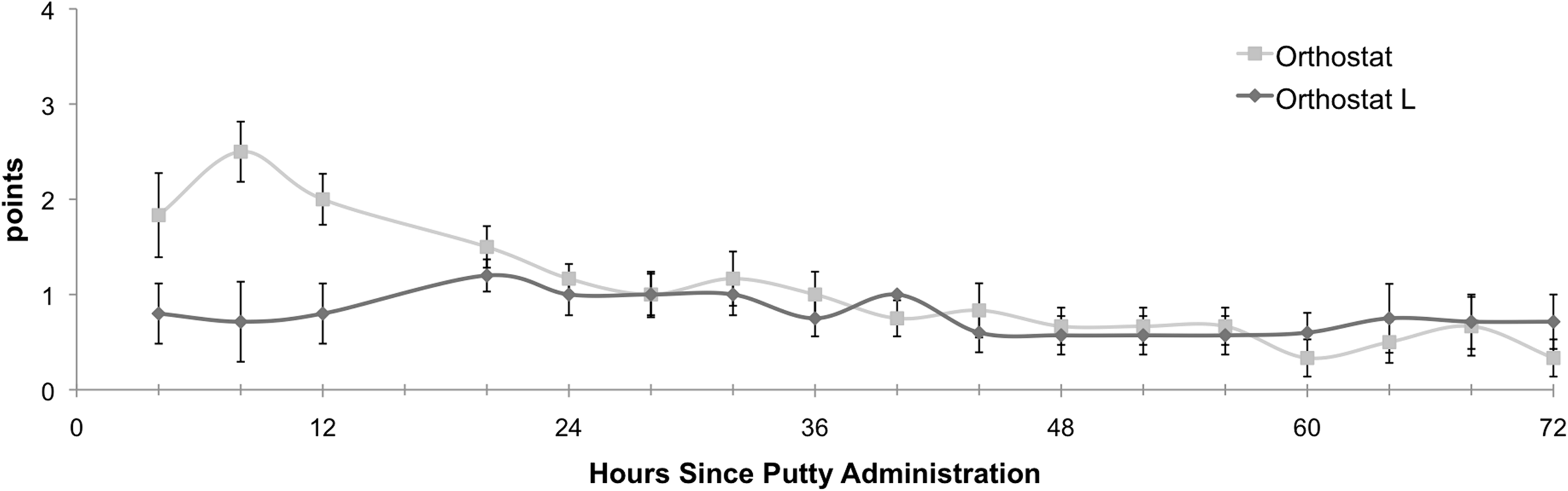

Supplement: Supplementary file 2 — Authors’ original file for figure 2 [file 12891_2013_2349_MOESM2_ESM.tiff]

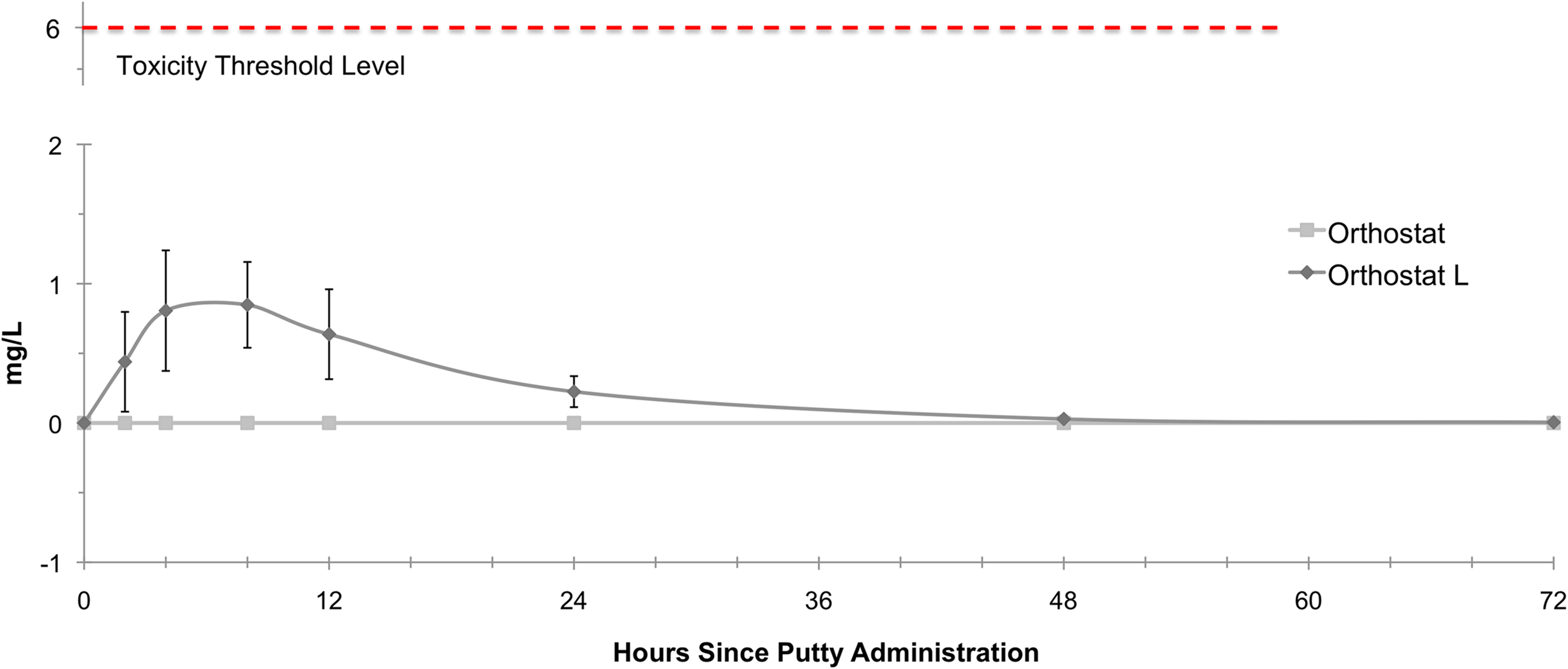

Supplement: Supplementary file 3 — Authors’ original file for figure 3 [file 12891_2013_2349_MOESM3_ESM.tiff]
